# Roles of octopaminergic and dopaminergic neurons in appetitive and aversive memory recall in an insect

**DOI:** 10.1186/1741-7007-7-46

**Published:** 2009-08-04

**Authors:** Makoto Mizunami, Sae Unoki, Yasuhiro Mori, Daisuke Hirashima, Ai Hatano, Yukihisa Matsumoto

**Affiliations:** 1 Graduate School of Life Sciences, Tohoku University, Katahira 2-1-1, Aoba-ku, Sendai 980-8577, Japan

## Abstract

**Background:**

In insect classical conditioning, octopamine (the invertebrate counterpart of noradrenaline) or dopamine has been suggested to mediate reinforcing properties of appetitive or aversive unconditioned stimulus, respectively. However, the roles of octopaminergic and dopaminergic neurons in memory recall have remained unclear.

**Results:**

We studied the roles of octopaminergic and dopaminergic neurons in appetitive and aversive memory recall in olfactory and visual conditioning in crickets. We found that pharmacological blockade of octopamine and dopamine receptors impaired aversive memory recall and appetitive memory recall, respectively, thereby suggesting that activation of octopaminergic and dopaminergic neurons and the resulting release of octopamine and dopamine are needed for appetitive and aversive memory recall, respectively. On the basis of this finding, we propose a new model in which it is assumed that two types of synaptic connections are formed by conditioning and are activated during memory recall, one type being connections from neurons representing conditioned stimulus to neurons inducing conditioned response and the other being connections from neurons representing conditioned stimulus to octopaminergic or dopaminergic neurons representing appetitive or aversive unconditioned stimulus, respectively. The former is called 'stimulus-response connection' and the latter is called 'stimulus-stimulus connection' by theorists studying classical conditioning in higher vertebrates. Our model predicts that pharmacological blockade of octopamine or dopamine receptors during the first stage of second-order conditioning does not impair second-order conditioning, because it impairs the formation of the stimulus-response connection but not the stimulus-stimulus connection. The results of our study with a cross-modal second-order conditioning were in full accordance with this prediction.

**Conclusion:**

We suggest that insect classical conditioning involves the formation of two kinds of memory traces, which match to stimulus-stimulus connection and stimulus-response connection. This is the first study to suggest that classical conditioning in insects involves, as does classical conditioning in higher vertebrates, the formation of stimulus-stimulus connection and its activation for memory recall, which are often called cognitive processes.

## Background

Insects are useful models for the study of cellular and molecular mechanisms of learning [1-4]. There is evidence suggesting that aminergic neurons convey reinforcing signals in classical conditioning in insects [5-15], as in mammals [16], and it has been suggested that octopaminergic (OA-ergic) and dopaminergic (DA-ergic) neurons convey reward and punishment signals, respectively (but see [17,18]). In honey bees, for example, Hammer [5] suggested that a putative OA-ergic neuron, VUMmx1 neuron (ventral unpaired median neuron of the maxillary neuromere in the subesophageal ganglion), mediates reinforcing properties of sucrose unconditioned stimulus (US) in appetitive olfactory conditioning. In the cricket *Gryllus bimaculatus*, we have shown that pharmacological blockade of octopamine (OA) receptors impairs conditioning of olfactory, visual pattern or color stimuli with water reward, whereas blockade of dopamine (DA) receptors specifically impairs conditioning of these stimuli with sodium chloride punishment [13-15].

The roles of OA and DA for memory recall in insects, however, have remained controversial. In fruit-flies, disruption of DA-ergic synaptic transmission had no effects on memory recall after aversive olfactory conditioning [7]. In honey bees, in contrast, Farooqui *et al*. [8] reported that disruption of OA-ergic transmission in the antennal lobe, the primary olfactory center and one of the termination areas of the VUMmx1 neuron, by an OA receptor antagonist (mianserin) or by RNA interference of the OA receptor gene impaired appetitive olfactory memory recall. Considering the observation by Hammer [5] that the VUMmx1 neuron was activated in response to olfactory conditioned stimulus (CS) after conditioning with sucrose US, Farooqui *et al*. [8] argued that activation of the VUMmx1 neuron by olfactory CS is required for recall of appetitive olfactory memory. The results of the study by Farooqui *et al*. [8], however, are not conclusive because the possibility that these treatments impaired memory consolidation or maintenance has not been excluded.

Here we show that OA and DA receptor antagonists impair appetitive and aversive memory recall, respectively, in olfactory and visual pattern conditioning in crickets. In order to account for this finding, we propose a new model of insect classical conditioning that assumes the involvement of two memory traces, one characterized as 'stimulus-stimulus connection' (S-S connection) and the other characterized as 'stimulus-response connection' (S-R connection) following the terminology of theorists studying classical conditioning in higher vertebrates [19-23], in contrast to previous models of insect classical conditioning [7] that are characterized as S-R connection models. We examined the validity of our model by pharmacological experiments using a cross-modal second-order conditioning procedure, and the results obtained fully supported the model. We suggest, for the first time, that the formation of S-S connection by conditioning and its activation for memory recall, which have often been referred to as cognitive processes in reports on classical conditioning in higher vertebrates [19-23], underlie classical conditioning in insects.

## Results

### OA and DA receptor antagonists impair the recall of appetitive and aversive olfactory memory, respectively

**Figure 1:**
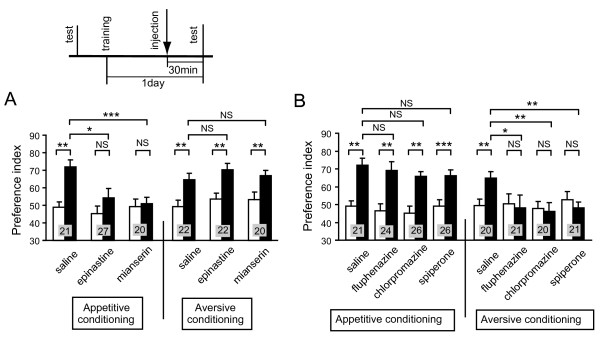
**Octopamine and dopamine receptor antagonists impair appetitive and aversive olfactory memory recall, respectively**. **(A, B) **Effects of octopamine (OA) (A) or dopamine (DA) (B) receptor antagonists on olfactory memory recall were studied. Twelve groups of animals were each subjected to two-trial appetitive (left) or six-trial aversive (right) olfactory conditioning trials. On the next day, each group was injected with 3 μl of saline or saline containing 1 μM epinastine, 1 μM mianserin, 500 μM fluphenazine, 500 μM chlorpromazine or 500 μM spiperone at 30 min before the final test. Preference indexes for rewarded odor (in the case of appetitive conditioning) or unpunished control odor (in the case of aversive conditioning) before (white bars) and after (black bars) conditioning are shown with means + SEM. The number of animals is shown at each data point in this and in all subsequent Figures. The results of statistical comparison before and after conditioning (Wilcoxon's test) and between experimental and saline-injected control groups (Mann-Whitney test) are shown as asterisks (*P < 0.05; ** *P *< 0.01; ***P < 0.001; NS *P *> 0.05).

First, the effects of epinastine and mianserin, antagonists of insect OA receptors, on the recall of appetitive olfactory memory were studied. The specificity of these drugs for insect neural OA receptors has been demonstrated in biochemical studies [24,25]. Three groups of animals were subjected to appetitive conditioning trials to associate an odor with water reward, using the procedure described previously [13]. One day after conditioning, the groups were each injected with physiological saline or saline containing epinastine or mianserin into the head hemolymph. The doses of drugs were based on results of our previous studies [13-15] and are stated in the Figure legends. The odor preference of animals was tested before conditioning and at 30 min after injection. The saline-injected control group exhibited a significant level of memory recall, that is, a significantly increased preference for the rewarded odor compared with that before conditioning (Figure 1A, left; for statistics, see legends), the level of which was as high as that in groups of intact animals we reported previously [13]. In contrast, the epinastine-injected and mianserin-injected groups exhibited no significantly increased preference for the rewarded odor (Figure 1A, left). This impairment may be due to impairment of (1) consolidation of memory, (2) maintenance of consolidated memory or (3) memory retrieval (recall). The first possibility is less likely because we have shown that protein-synthesis dependent long-term memory is fully established 12 hours after conditioning [26] and thus consolidation is likely to be completed by this time. To examine the second possibility, animals in one control group were subjected to appetitive conditioning trials and were injected with epinastine one day later and then tested one day after injection, at which time epinastine had been fully metabolized. They exhibited a significantly increased preference for the rewarded odor [see Additional file 1A, left]. The finding that animals exhibit impaired learning performance only under the influence of OA receptor antagonists ruled out the second possibility. Thus we conclude that OA receptor antagonists impair recall of appetitive olfactory memory.

Next, the effects of OA receptor antagonists on the recall of aversive olfactory memory were studied. Three groups of animals were subjected to aversive conditioning trials to associate an odor with 20% sodium chloride solution. One day later, the groups were each injected with saline or saline containing epinastine or mianserin and were subjected to retention tests at 30 min after injection. The groups injected with epinastine and mianserin exhibited significantly increased preferences for the control odor (and thus significantly decreased preference for the punished odor), the levels of preferences being as high as that of the saline-injected control group (Figure 1A, right). Thus, OA receptor antagonists do not impair aversive memory recall. This finding also suggests that OA receptor antagonists do not impair sensory or motor functions or the motivation necessary for normal learning performance.

We also found that fluphenazine, chlorpromazine and spiperone, antagonists of insect DA receptors, impair aversive olfactory memory recall. The specificity of these drugs for insect neural OA receptors has been examined in biochemical studies [27,28]. Four groups of animals were subjected to aversive conditioning trials. One day later, they were each injected with saline or saline containing fluphenazine, chlorpromazine, or spiperone and were subjected to retention tests at 30 min after injection. The groups injected with fluphenazine, chlorpromazine and spiperone exhibited complete impairment of memory recall: they exhibited no significantly decreased preference for the punished odor (Figure 1B, right). In contrast, the saline-injected control group exhibited significantly decreased preference for the punished odor (Figure 1B, right). The impairment in the experimental group was not due to impairment of memory maintenance, since animals in the group that received aversive conditioning trials and were injected with fluphenazine one day later and tested one day after injection exhibited no impairment of memory recall [see Additional file 1A, left].

DA receptor antagonists had no effects on appetitive olfactory memory recall. Four groups of animals were subjected to appetitive conditioning trials. One day later, they were each injected with saline or saline containing fluphenazine, chlorpromazine or spiperone and tested at 30 min after injection. The groups injected with fluphenazine, chlorpromazine and spiperone exhibited no impairment of appetitive memory recall (Figure 1B, left). This finding also suggests that DA receptor antagonists do not impair sensory or motor functions or the motivation necessary for normal learning performance.

Because the effects of DA and OA receptor antagonists on memory recall were studied after two-trial appetitive conditioning and six-trial aversive conditioning, it could be argued that different effects of OA and DA receptor antagonists are ascribed to the difference in the number of trials. However, this is unlikely because we have observed that memory recall after six-trial appetitive conditioning is fully impaired by epinastine (data not shown).

The impairment of appetitive memory recall and aversive memory recall by injection of OA and DA receptor antagonists, respectively, indicates that intact synaptic transmission from OA- and DA-ergic neurons is necessary for appetitive memory recall and aversive memory recall, respectively. Impairment of the recall of appetitive memory and aversive memory by blockade of OA and DA receptors, respectively, was not specific to 1-day (long-term) memory but was also found for 1-h (mid-term) memory [see Additional file 2].

### OA and DA receptor antagonists impair the recall of appetitive and aversive visual memory, respectively

**Figure 2:**
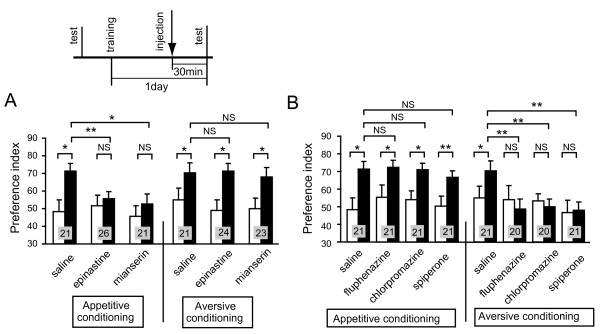
**Octopamine and dopamine receptor antagonists impair appetitive and aversive visual memory recall, respectively**. **(A, B) **Effects of octopamine (OA) (A) or dopamine (DA) (B) receptor antagonists on visual memory recall were studied. Twelve groups of animals were each subjected to 8-trial appetitive (left) or 12-trial aversive (right) conditioning trials. On the next day, each group was injected with 3 μl of saline or saline containing 1 μM epinastine, 1 μM mianserin, 500 μM fluphenazine, 500 μM chlorpromazine or 500 μM spiperone at 30 min before the final test. Preference indexes for rewarded odor (in the case of appetitive conditioning) or unpunished control odor (in the case of aversive conditioning) before (white bars) and after (black bars) conditioning are shown with means + SEM. The results of statistical comparison before and after conditioning (Wilcoxon's test) and between experimental and saline-injected control groups (Mann-Whitney test) are shown as asterisks (* *P *< 0.05; ** *P *< 0.01; NS *P *> 0.05).

We next investigated whether the finding that OA and DA receptor antagonists specifically impair the recall of appetitive memory and aversive memory, respectively, is applicable to the recall of visual memory. First, the effects of an OA receptor antagonist on the recall of appetitive visual memory were studied. Three groups of animals were subjected to appetitive conditioning of a visual pattern, using the procedure described previously [14]. One day later, they were each injected with saline or saline containing epinastine or mianserin and subjected to retention tests at 30 min after injection. The groups injected with epinastine and mianserin exhibited no significant level of memory recall (Figure 2A, left), whereas the saline-injected control group exhibited a significant level of memory recall (Figure 2A, left). The impairment was again not due to impairment of memory maintenance, because animals in another control group that received appetitive conditioning trials and were injected with epinastine 1 day after conditioning and tested 1 day after injection exhibited no impairment of memory recall [see Additional file 1B, left].

Next, the effects of an OA receptor antagonist on the recall of aversive visual memory were studied. Another three groups of animals were subjected to aversive conditioning trials. One day later, the groups were each injected with saline or saline containing epinastine or mianserin and tested at 30 min after injection. The groups injected with epinastine and mianserin exhibited significant levels of memory recall, indicating that an OA receptor antagonist does not impair aversive memory recall (Figure 2A, right). Thus, blockade of OA receptors impairs appetitive visual memory recall without affecting aversive visual memory recall.

Similarly, injection of DA receptor antagonists impaired aversive visual memory recall (Figure 2B, right). Four groups of animals were subjected to aversive visual conditioning trials. One day later, the groups were each injected with saline or saline containing fluphenazine, chlorpromazine or spiperone and subjected to retention tests at 30 min after injection. The groups injected with fluphenazine, chlorpromazine and spiperone exhibited complete impairment of memory recall, although the saline-injected group exhibited a significant level of memory recall (Figure 2B, right). The impairment was not due to impairment of memory maintenance, because animals in the control group that received aversive conditioning trials and were injected with epinastine 1 day after conditioning and tested 1 day after injection exhibited no impairment of memory recall [see Additional file 1B, right].

Injection of DA receptor antagonists had no effect on appetitive visual memory recall. Four groups of animals were subjected to appetitive visual conditioning. One day later, the groups were each injected with saline or saline containing fluphenazine, chlorpromazine or spiperone and tested at 30 min after injection. The groups injected with fluphenazine, chlorpromazine and spiperone exhibited significant levels of memory recall, the levels of which were as high as the level in the saline-injected group (Figure 2B, left). Thus, we conclude that intact synaptic transmission from DA-ergic neurons is needed for aversive visual memory recall but not for appetitive visual memory recall.

### Re-examination of the effect of an OA receptor antagonist on visual conditioning

We previously concluded that OA/DA receptor antagonists impaired olfactory and visual pattern conditioning on the basis of observations that groups of crickets injected with these drugs 30 min before conditioning exhibited no conditioning effects, that is, no significantly changed preferences for the conditioned stimuli, in retention tests performed 1 day (for olfactory conditioning) or 30 min (for visual pattern conditioning) after completing the conditioning [13,14]. The present finding that these drugs impair olfactory and visual pattern memory recall, however, prompted us to re-examine the validity of this conclusion, because if the effect of drugs remained during the retention test, the impairment may not be due to an impairment of conditioning.

It is obvious that our conclusion that an OA antagonist and a DA antagonist impair appetitive and aversive olfactory conditioning is valid, because the effects of epinastine (OA receptor antagonist) and fluphenazine (DA receptor antagonist) do not last for 1 day [see Additional files 1, 2] [13] and thus it is unlikely that effects of these drugs remained during the retention test, which was performed 1 day after conditioning.

In the case of visual pattern conditioning, however, our conclusion can be justified only for aversive conditioning. We observed that fluphenazine impaired aversive olfactory conditioning when it was injected 30 min before conditioning but not when it was injected 60 min before conditioning [13]. The retention test after aversive visual pattern conditioning started 120 min after fluphenazine injection [14] and thus it is unlikely that the effect of fluphenazine remained during the retention test. We observed, however, that epinastine was effective when it was injected 120 min before olfactory conditioning [13], and the retention test after appetitive visual conditioning was performed 100 min after epinastine injection. Thus, it is most likely that the effects of epinastine remained during testing.

**Figure 3:**
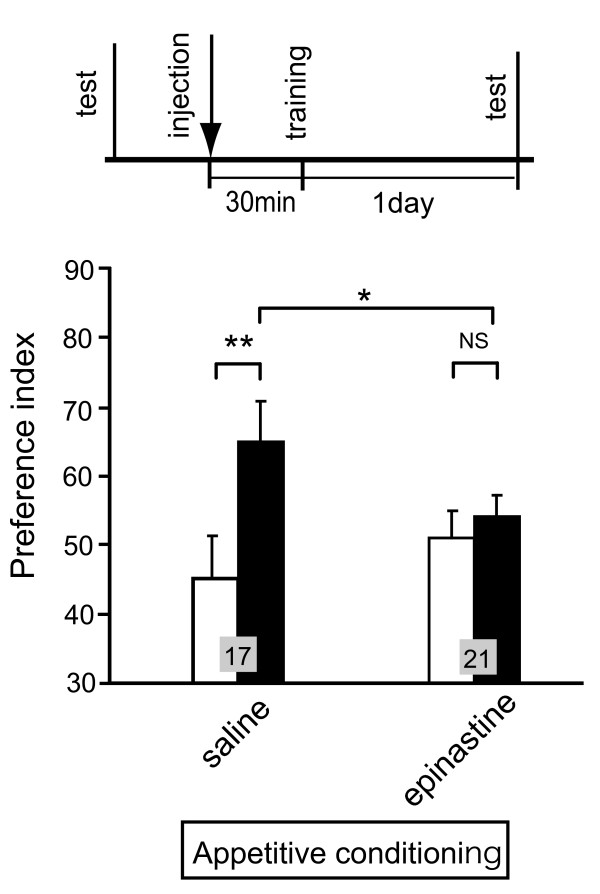
**Effects of epinastine on appetitive visual pattern conditioning**. Two groups of animals were injected with 3 μl of saline or saline containing 1 μM epinastine 30 min before appetitive visual pattern conditioning. The retention test was performed 1 day after conditioning. Preference indexes for rewarded pattern before (white bars) and after (black bars) conditioning are shown with means + SEM. The results of statistical comparison before and after conditioning (Wilcoxon's test) and between experimental and saline-injected control groups (Mann-Whitney test) are shown as asterisks (* *P *< 0.05; ** *P *< 0.01; NS *P *> 0.05).

We performed, therefore, new experiments to re-examine the effect of epinastine on appetitive visual pattern conditioning. A group of animals was injected with epinastine 30 min before appetitive visual pattern conditioning and subjected to retention tests at 1 day after conditioning. The group exhibited no significantly increased preference for the rewarded pattern (Figure 3), thus indicating complete impairment of conditioning. We conclude that intact synaptic transmission from OA-ergic neurons is needed for appetitive visual pattern conditioning.

### Proposal of a novel model of insect classical conditioning

**Figure 4:**
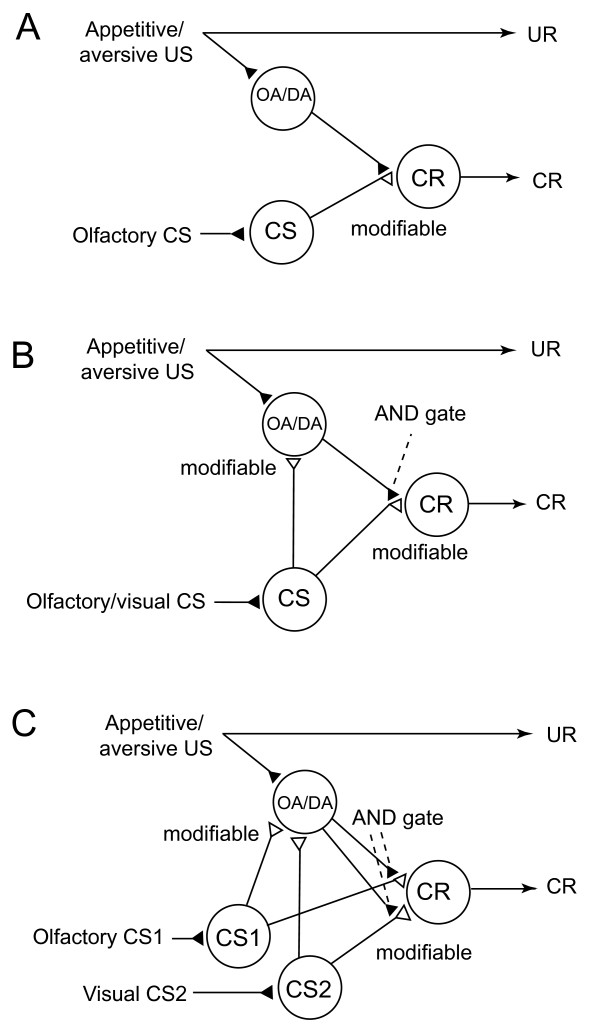
**Neural models of classical conditioning in insects**. **(A) **A model proposed to account for the roles of intrinsic and extrinsic neurons of the mushroom body in olfactory conditioning in fruit-flies [5]. Octopaminergic or dopaminergic neurons ('OA/DA' neurons) convey signals for appetitive or aversive unconditioned stimulus, respectively. 'CS' neurons, which convey signals for CS, make synaptic connections with 'CR' neurons that induce conditioned response (CR), the efficacy of the connection being strengthened by conditioning. 'OA/DA' neurons make synaptic connections with presynaptic terminals of 'CS' neurons. **(B) **A new model of classical conditioning, termed Mizunami-Unoki or M-U model. The model assumes that efficacy of synaptic transmission from 'CS' neurons to 'OA/DA' neurons is strengthened by conditioning and that coincident activation of 'OA/DA' neurons and 'CS' neurons is needed to activate 'CR' neurons to lead to a CR (AND gate). **(C) **M-U model to account for second-order conditioning.

Notably, our finding that intact synaptic transmission from OA- and DA-ergic neurons is needed for the recall of appetitive memory and aversive memory, respectively, is not consistent with conventional neural models of insect classical conditioning. Figure 4A depicts perhaps the best model proposed to account for the roles of extrinsic and intrinsic neurons of the mushroom body, a higher-order association center of the insect brain, in olfactory conditioning in the fruit-fly *Drosophila *[7]. In this model, it is postulated that (1) 'CS' neurons (intrinsic neurons of the mushroom body) that convey signals about a CS make synaptic connections with dendrites of 'CR' neurons (efferent neurons of the lobes of the mushroom body), activation of which leads to a conditioned response (CR) that mimics unconditioned response (UR), but these synaptic connections are silent or very weak before conditioning; (2) OA- or DA-ergic efferent neurons projecting to the lobes ('OA/DA' neurons), which convey signals for appetitive or aversive US, respectively, make synaptic connections with axon terminals of 'CS' neurons, and (3) the efficacy of the synaptic connection from 'CS' neurons to 'CR' neurons that induce a CR (CS-CR or S-R connection) is strengthened by coincident activation of 'CS' neurons and 'OA/DA' neurons during conditioning (Figure 4A). This model assumes that presentation of a CS after conditioning activates the CS-CR or S-R connection to induce a CR. This model is characterized as an S-R model, following terminology in studies on classical conditioning in higher vertebrates [19-23]. This model is in accordance with our previous findings that intact synaptic output from OA- or DA-ergic neurons is needed to achieve appetitive or aversive conditioning, respectively ([13-15]; Figure 3 of the present study) but does not account for the present finding that it is also needed to achieve appetitive or aversive memory recall, respectively.

We propose a new model (Figure 4B) with minimal modifications of the model by Schwaerzel *et al*. [7], by considering the present findings in crickets and previous findings in honey bees by Hammer [5] and Farooqui *et al*. [8] described in the introductory section. We assume, at first, that activation of 'OA/DA' neurons and resulting release of OA or DA are needed to 'gate' the sensori-motor pathway from the 'CS' neurons to 'CR' neurons (Figure 4B) after conditioning. Secondly, in order to account for the activation of OA/DA neurons during memory recall, we assume that synaptic connection from 'CS' neurons to 'OA/DA' neurons representing unconditioned stimulus (US), which is termed CS-US or S-S connection [19-23], is strengthened by coincident activation of 'CS' neurons and 'OA/DA' neurons during conditioning. In short, our model assumes the formation of two kinds of memory traces by conditioning and activation of both kinds for memory recall. This model is characterized as a hybrid of the S-S and S-R models.

An alternative possibility to explain our finding is that different sets of 'OA/DA' neurons govern reinforcement and memory retrieval processes, respectively. This is achieved by modifying the model shown in Figure 4A by assuming other 'OA/DA' neurons in neural pathways downstream of the 'CR' neurons. It is difficult, however, for this model to account for our results with second-order conditioning described below.

**Figure 5:**
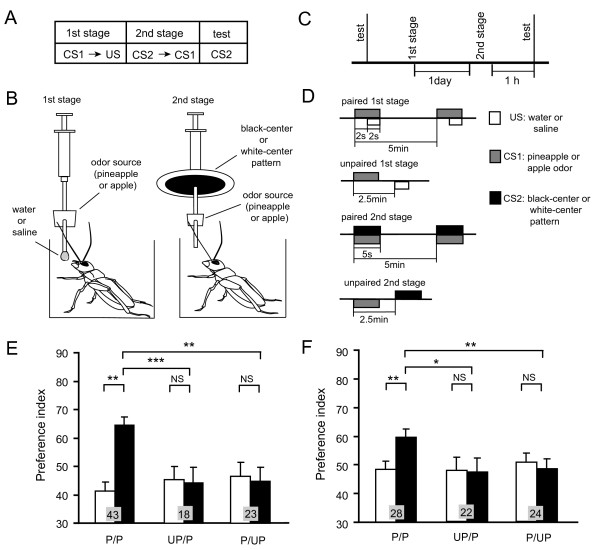
**Second-order conditioning**. **(A-C) **General scheme (A), conditioning procedure (B), and time schedule (C) for appetitive and aversive second-order conditioning. **(D) **Stimulus schedules in the second-order conditioning group are initial paired presentation of CS1 and unconditioned stimulus (US) and subsequent paired presentations of CS2 and CS1 (paired/paired (P/P) group), and those in control groups are unpaired presentations in the first or second conditioning stage (unpaired/paired (UP/P) or paired/unpaired (P/UP) groups). **(E, F) **Two groups of animals were each subjected to appetitive (E) or aversive (F) second-order conditioning trials (P/P groups). Four control groups were each subjected to unpaired presentations in the first (UP/P groups) or second (P/UP groups) stage in appetitive (E) or aversive (F) second-order conditioning. Animals received four first-stage trials and then four second-stage trials for appetitive second-order conditioning, and six first-stage trials and then four second-stage trials for aversive second-order conditioning. Preference indexes for the CS2 (in the case of appetitive second-order conditioning) or control pattern (in the case of aversive second-order conditioning) before (white bars) and after (black bars) conditioning are shown with means + SEM. The results of statistical comparison are shown as asterisks (* *P *< 0.05; ** *P *< 0.01; *** *P *< 0.001; NS *P *> 0.05).

The core of our model is that a 'CS-OA/DA' pathway (S-S connection) is formed during conditioning. We evaluated this assumption by using a second-order conditioning procedure. Second-order conditioning is a technique for testing whether a stimulus can acquire the reinforcing property of a US by conditioning with the US and the stimulus can support new conditioning thereafter. In actual experiment, a stimulus (CS1) is paired with a US and then a new stimulus (CS2) is paired with the CS1 (Figure 5A). In our model, second-order conditioning is accounted for by the formation a 'CS-OA/DA' pathway in the initial CS1-US pairing stage, which allows the CS1 to activate OA- or DA-ergic neurons and thus to substitute for the US in the second CS2-CS1 pairing stage (Figure 4C). A notable prediction from our model is that blockade of OA or DA receptors during the initial CS1-US pairing stage does not impair the enhancement of synapses from 'CS' neurons to 'OA/DA' neurons. This is because blockade of OA or DA receptors (of 'CR' neurons) should not affect normal activities of 'CS' neurons and 'OA/DA' neurons. In other words, impairment of normal functioning of OA/DA receptors should impair establishing one kind of memory traces (S-R connection) but not establishing the other (S-S connection). Hence, a stimulus that has been conditioned with a US under the blockade of OA or DA receptors does not induce a CR [13-15], but the stimulus should still be able to support new conditioning. Therefore, our model predicts that blockade of OA or DA receptors during the first CS1-US pairing stage does not impair second-order conditioning. In contrast, the same treatment during the second CS2-CS1 pairing stage or during the final retention test should impair second-order conditioning or memory recall, respectively.

### Second-order conditioning in crickets

Second-order conditioning has been reported in honey bees [29,30] and fruit-flies [31] but not in crickets. Therefore, we first studied the capability of crickets to achieve second-order conditioning. We used an olfactory stimulus as CS1 and a visual pattern as CS2 (Figure 5B): such cross-modal second-order conditioning has not been reported in any species of insects. The second-order conditioning procedure and control procedure are depicted in Figure 5C and 5D. A group of animals that was subjected to appetitive second-order conditioning trials exhibited significantly increased preference for the CS2 (Figure 5E). In contrast, control groups that were each subjected to unpaired presentations of stimuli at the first or second conditioning stage exhibited no significantly increased preference for the CS2 (Figure 5E), thus indicating that the increased preference for the CS2 in the experimental group is truly the result of second-order conditioning. Another group of animals that was subjected to aversive second-order conditioning trials also exhibited significantly increased preference for the control pattern (and thus significantly decreased preference for the CS2) (Figure 5F). Control groups that were each subjected to unpaired presentations of stimuli at the first or second conditioning stage exhibited no significantly increased preference for the control pattern (Figure 5F), thus indicating that the decreased preference for the CS2 observed in the experimental group is truly the result of aversive second-order conditioning. The aversive second-order conditioning, however, was less effective than the appetitive second-order conditioning (see Figure 5E).

### Evaluation of our model by using the second-order conditioning procedure

**Figure 6:**
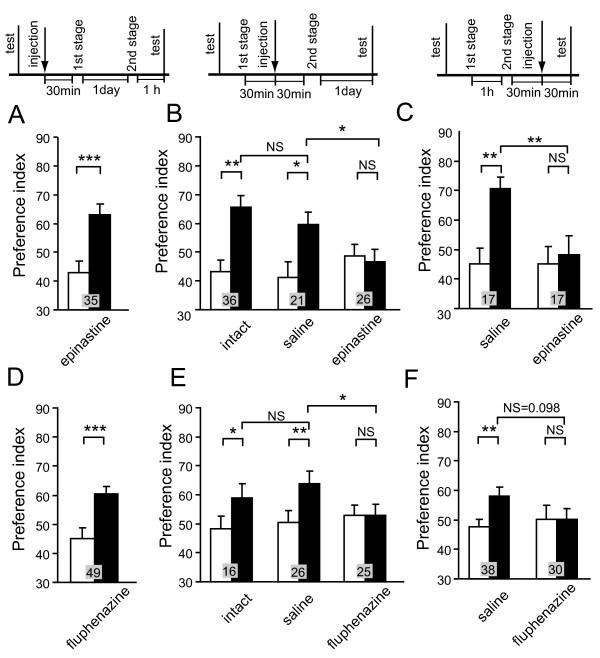
**Octopamine and dopamine receptor antagonists impair appetitive and aversive second-order conditioning**. **(A-C) **Three groups of animals were each injected with 3 μl of saline containing 1 μM epinastine at 30 min before the first conditioning stage (A), before the second conditioning stage (B), or before the final test (C) in appetitive second-order conditioning. One control group received no injection (B, intact), and two other groups were each injected with saline at 30 min before the second conditioning stage (B, saline), or before the final test (C, saline). **(D-F) **Three groups were each injected with 3 μl of saline containing 500 μM fluphenazine at 30 min before the first conditioning stage (D), before the second conditioning stage (E), or before the final test (F) in aversive second-order conditioning. Animals received four first-stage trials and then four second-stage trials for appetitive second-order conditioning, and six first-stage trials and then four second-stage trials for aversive second-order conditioning. One control group received no injection (E, intact), and two other groups were each injected with saline at 30 min before the second conditioning stage (E, saline) or before the final test (F, saline). Preference indexes for the CS2 (in the case of appetitive second-order conditioning) or the control pattern (in the case of aversive second-order conditioning) before (white bars) and after (black bars) conditioning are shown with means + SEM. The results of statistical comparison are shown as asterisks (* *P *< 0.05; ** *P *< 0.01; *** *P *< 0.001; NS *P *> 0.05).

We then proceeded to the evaluation of our model by using the second-order conditioning procedure. First, we studied the effects of injection of an OA receptor antagonist before the initial stage of appetitive second-order conditioning. One group of animals was injected with saline containing epinastine, and 30 min later the group was subjected to the first appetitive conditioning stage in which CS1 was paired with an appetitive US (Figure 6A). The second CS1-CS2 pairing stage was performed 1 day after completion of the first conditioning stage, assuring that epinastine had been fully metabolized at this stage. The final test was performed 1 h after completion of the second conditioning stage. The group injected with epinastine before the first conditioning stage exhibited a significantly increased preference for the CS2, thus indicating that appetitive second-order conditioning is achieved (Figure 6A).

In contrast, epinastine impaired appetitive second-order conditioning when it was injected before the second conditioning stage. Two groups of animals were subjected to the first conditioning stage and each group was injected with saline or saline containing epinastine 30 min later. They were subjected to the second conditioning stage 30 min after the injection. The test was performed 1 day after completion of the second conditioning stage, assuring that epinastine had been fully metabolized at that time. Here, the time schedule was slightly changed from that shown in Figures 5E and 6A in order to make the schedule as short as possible so as to assure that the animals remain healthy. Therefore, we confirmed in another group of animals that second-order conditioning is achieved with this changed time schedule (Figure 6B, intact). The group injected with saline before the second conditioning stage exhibited a significantly increased preference for the CS2, the level being as high as that in a group of intact animals (Figure 6B). In contrast, the group injected with epinastine before the second conditioning stage exhibited no significantly increased preference for the CS2 (Figure 6B, epinastine), thus indicating complete impairment of second-order conditioning.

Epinastine also impaired the recall of memory formed by appetitive second-order conditioning. Two groups of animals were subjected to appetitive second-order conditioning and each group was injected with saline or saline containing epinastine 30 min later. The final test was performed 30 min after the injection. The control group injected with saline before the test exhibited a significantly increased preference for the CS2. In contrast, the group injected with epinastine before the final test exhibited no significantly increased preference for the CS2 (Figure 6C). The impairment was not due to impairment of memory maintenance, because animals in the group that received appetitive second-order conditioning trials and were injected with epinastine at 30 min after completion of the second conditioning stage and tested 1 day after injection exhibited a significant level of memory recall [see Additional file 3].

Next, we studied the effects of injection of a DA receptor antagonist before the initial stage of aversive second-order conditioning. A group of animals was injected with fluphenazine, and 30 min later the group was subjected to the first aversive conditioning stage to associate CS1 with aversive US. One day later, the group was subjected to the second CS2-CS1 pairing stage, and the final test was performed 1 h after the second conditioning stage. The group injected with fluphenazine before the first conditioning stage exhibited a significantly increased preference for the control pattern (and thus significantly decreased preference for the CS2) (Figure 6D), thus indicating that aversive second-order conditioning is achieved.

In contrast, fluphenazine impaired aversive second-order conditioning when it was injected before the second conditioning stage. Two groups were subjected to the first conditioning stage and each group was injected with saline or saline containing fluphenazine 30 min later. At 30 min after injection, they were subjected to the second conditioning stage. The final test was performed 1 day after completion of the second conditioning stage. We showed in another group of animals that aversive second-order conditioning could be achieved with this time schedule (Figure 6E, intact). The saline-injected group exhibited a significantly increased preference for the control pattern, the level of the preference being as high as that of intact animals (Figure 6E). In contrast, the group injected with fluphenazine before the second conditioning stage exhibited no significantly increased preference for the control pattern, thus indicating complete impairment of second-order conditioning.

Fluphenazine also impaired the recall of memory formed by aversive second-order conditioning. Two groups were subjected to aversive second-order conditioning trials. The groups were injected with saline or saline containing fluphenazine at 30 min after completion of the conditioning trials and tested at 30 min after injection. The control group injected with saline before the test exhibited a significantly increased preference for the control pattern (Figure 6F). In contrast, the group injected with fluphenazine before the final test exhibited no significantly increased preference for the control pattern (Figure 6F). This impairment was not due to impairment of memory maintenance, because animals in the group that received aversive second-order conditioning trials and were injected with fluphenazine at 30 min after completion of the conditioning trials and tested 1 day after injection exhibited a significantly increased preference for the control pattern [see Additional file 3]. We thus conclude that fluphenazine impairs memory recall. We observed, however, that the significance of the difference between the saline-injected group and the fluphenazine-injected group was marginal (*P *= 0.098), probably because aversive second-order conditioning trials yielded only a relatively low level of conditioning effect. On account of the imperfectness of data, the results are in good accordance with our model. It should be noted that alternative models in which different sets of OA/DA neurons participate in reinforcement and memory recall do not account for our finding, since these models predict an impairment of second-order conditioning when OA/DA receptors are blocked at any stage of conditioning.

## Discussion

### Major findings

We found, at first, that epinastine and mianserin, OA receptor antagonists, impair appetitive memory recall without affecting aversive memory recall, whereas fluphenazine, chlorpromazine, and spiperone, DA receptor antagonists, impair aversive memory recall without affecting appetitive memory recall, in olfactory and visual pattern learning in crickets. Although the specificity of some of antagonists used in this study is not perfect, as we have discussed previously [13], we observed in our previous studies [13-15] and in this study that different kinds of antagonists had the same effects, that is, all presumed OA or DA receptor antagonists impaired only appetitive or aversive conditioning and memory recall, respectively, suggesting that the observed effect is due to the blockade of OA or DA receptors. The results of this study suggest that intact synaptic transmission from OA- and DA-ergic neurons is needed for the recall of appetitive memory and aversive memory, respectively.

In order to account for this finding, we proposed a model with the assumptions that (1) two synaptic connections, that is, synaptic connection from neurons representing CS to OA/DA neurons representing US (CS-US or S-S connection in studies on classical conditioning in higher vertebrates [19-23]) and that from neurons representing CS to neurons that induce CR (S-R connection), are strengthened by conditioning and (2) activation of both connections is needed to induce CR (Figure 4B). In short, our model assumes that two kinds of memory traces are formed by conditioning and that both kinds need to be activated for memory recall. Our model predicts that, although injection of OA/DA receptor antagonists before first-order conditioning fully impaired conditioning ([13-15]; Figure 3 of the present study), injection of these drugs before the initial CS1-US pairing stage of the second-order conditioning does not impair second-order conditioning, because blockade of OA/DA receptors should not impair the growth of the CS1-US connection, which allows the CS1 to activate the OA/DA neurons and thus provides reinforcing properties to the CS1 at the second CS2-CS1 pairing stage. The results of our experiments with second-order conditioning fully supported this prediction.

### Comparisons with reports on other species of insects

Our finding that OA-ergic signaling and DA-ergic signaling are needed for appetitive and aversive memory recall, respectively, is in accordance with some previous findings but not with others. In honey bees, Hammer [5] reported that a putative OA-ergic neuron, called VUMmx1 neuron, mediates reinforcing properties of sucrose US and that this neuron exhibited increased responses to olfactory CS after conditioning with sucrose US. Moreover, based on the results of a study using an OA receptor antagonist, mianserin, and RNA interference of expression of the OA receptor gene, Farooqui *et al*. [8] suggested that normal OA-ergic synaptic transmission in the antennal lobe, one of the termination areas of the VUMmx1 neuron, is needed for memory recall in appetitive olfactory conditioning with sucrose US. Farooqui *et al*. [8] argued that activation of the VUMmx1 neuron by olfactory CS is needed for appetitive memory recall, in full accordance with our model. In fruit-flies, it has also been reported that DA-ergic neurons innervating the mushroom body exhibited enhanced responses to olfactory CS after pairing olfactory CS with electric shock US [9], but it is not known whether the activation is needed for memory recall.

Results of other studies, however, are inconsistent with our findings. A study using transgenic fruit-flies has suggested that intact DA-ergic synaptic transmission is not needed for memory recall after pairing olfactory CS with electric shock US [7]. In honey bees, it has been reported that injection of OA in the antennal lobe or the calyx of the mushroom body can substitute, at least in part, presentation of sucrose US for achieving olfactory conditioning [6]. This finding is not consistent with our model, because it is obvious that memory recall after this substitutive conditioning does not accompany an activation of the VUMmx1 neuron, since injection of OA does not activate the VUMmx1 neuron and thus it is unlikely that synaptic connection for the olfactory CS to activate the VUMmx1 neuron is enhanced by pairing of CS with OA injection, if enhancement of the synaptic transmission requires coincident activation of the pre- and post-synaptic neurons. Thus, these findings are consistent with the view that activation of OA- or DA-ergic neurons by the CS is not needed for memory recall, thereby supporting conventional S-R models.

Based on our findings in crickets and those reported in other species of insects, we propose that activations of OA/DA neurons are needed for memory recall in some forms of classical conditioning in insects but not in other forms of classical conditioning. Such diversity is noted in classical conditioning in higher vertebrates as is discussed below. The critical factors for the requirement of OA/DA-ergic signaling in appetitive/aversive memory recall in insect classical conditioning should be determined.

Second-order conditioning has been reported in honey bees [29,30] and fruit-flies [31], but this study is the first to demonstrate cross-modal second-order conditioning, in which olfactory stimulus was used as CS1 and visual stimulus was used as CS2. Successful demonstration of cross-modal second-order conditioning indicates that OA/DA-ergic neurons that convey reward/punishment signals in olfactory learning can also convey reward/punishment signals in visual learning. This finding supports our proposal [14,15] that OA/DA-ergic neurons serve as general reward/punishment systems, conveying reinforcement signals in the learning of a variety of sensory signals.

An important future subject is to determine the sites of convergence of CS and US for olfactory and visual pattern learning in crickets. In fruit-flies [1,32] and honey bees [4,33,34], the antennal lobe and the mushroom bodies have been considered as sites of convergence of olfactory CS and US in olfactory conditioning. In the cricket *Acheta domesticus*, the mushroom body has been suggested to participate in olfactory learning [35]. In fruit-flies, a recent study has suggested that the central complex participates in visual pattern learning [36]. Thus, the focus of our study should be the antennal lobe, the mushroom bodies, and the central complex. Unfortunately, distributions of neurons immunoreactive to OA and DA in these brain areas have not been fully characterized in crickets [37] and studies are therefore needed on this subject. A more important subject is to determine the precise locations of two kinds of memory traces.

### Comparisons with classical conditioning in mammals

In higher vertebrates, two theories to account for classical conditioning have been proposed. In one theory, classical conditioning is viewed as the formation of a new reflex pathway for the CS to evoke a CR, as a result of pairing of the CS with a US (S-R theory [19-23]). According to this view, an initially insignificant event, CS, is incorporated into the reflex system under the control of a more biologically significant stimulus, US, whenever those two events occur in close temporal contiguity. This view accounts for some forms of classical conditioning in higher vertebrates [19-23]. Many other forms of classical conditioning in higher vertebrates, however, have been suggested to involve the formation of connection between neurons representing CS and those representing US (that is, S-S connection). According to this view (S-S theory), associations are formed between internal representation of the CS and that of the US, and the growth of this association permits the CS to activate a representation of the US in the absence of the US itself. This anticipatory activation of the US representation produces the CR. This view is also referred to as the cognitive account of classical conditioning [19-23], since it assumes the formation of internal representation of the relationship between external sensory events (that is, contingent occurrence of the CS and US) by conditioning and that its activation leads to memory recall. Our model involves both the S-R and S-S connections and is the first to propose that the S-S or cognitive account is applicable to classical conditioning in insects.

## Conclusion

We conclude that activation of OA- and DA-ergic neurons is necessary for appetitive and aversive memory recall, respectively, in crickets. We also suggest that the S-S or cognitive account of classical conditioning proposed for higher vertebrates is applicable to insects. Because the brain of insects, which we refers to as 'microbrain' [38,39], consists of relatively small numbers of neurons and is accessible to various kinds of experimental manipulation [1-4], insects should emerge as pertinent models for studying neural mechanisms of sophisticated information processing underlying classical conditioning.

## Methods

### Insects

Adult male crickets, *G. bimaculatus*, at 1 week after the imaginal molt were used. Three days before the start of the experiment, animals were deprived of drinking water to enhance their motivation to search for water.

### Olfactory conditioning procedure

We used classical conditioning and operant testing procedures described previously [13,14,40,41]. Banana or pineapple odor was used as CS, and water or saline (20% sodium chloride solution) was used as US. A syringe containing water and a syringe containing sodium chloride solution were used for appetitive conditioning and aversive conditioning, respectively. A filter paper soaked with banana or pineapple essence was attached to the needle of the syringe. The filter paper was placed above the cricket's head so as to present an odor, and then water reward or sodium chloride punishment was presented to the mouth for appetitive or aversive conditioning, respectively. After the conditioning trials, the air in the beaker was ventilated. The crickets were subjected to two pairing trials for appetitive conditioning and six pairing trials for aversive conditioning. The inter-trial interval (ITI) was 5 min.

### Odor preference test

The methods used for the test were described previously [13]. All groups of animals were subjected to odor preference tests before and after conditioning. On the floor of the test chamber of the test apparatus were two holes that connected the chamber with two odor sources. Each odor source consisted of a plastic container containing a filter paper soaked with 3 μl solution of banana or pineapple essence, covered with fine gauze net. Three containers were mounted on a rotative holder and two of three odor sources could be located simultaneously just below the holes of the test chamber. Before the odor preference test, a cricket was transferred to the waiting chamber at the waiting position and left for about 4 min to become accustomed to the surroundings. Then the cricket was allowed to enter the test chamber and the test started. Two min later, the relative positions of the banana and pineapple sources were changed by rotating the container holder. The preference test lasted for 4 min. If the total time of visits of an animal to either source was less than 10 s, we considered that the animal was less motivated to visit odor sources, possibly due to a poor physical condition, and the data were rejected. We found no significant difference in the proportion of animals that visited olfactory sources (or visual targets, see below) for less than 10 s between the saline-injected group and drug-injected group for any of the drugs used in this study. The groups in which banana or pineapple was used as CS exhibited no significantly different levels of conditioning effects, and thus the data from the two groups were pooled.

### Visual conditioning procedure

The procedure for visual conditioning was described previously [14]. A black-center and white-surround pattern (black-center pattern) or a white-center and black-surround pattern (white-center pattern) was used as CS and water or sodium chloride solution was used as US. A syringe containing water and a syringe containing sodium chloride solution were used for appetitive conditioning and aversive conditioning, respectively. A pattern was attached to the needle of the syringe. The pattern was presented above the cricket's head and then water reward or sodium chloride punishment was presented to the mouth for appetitive or aversive conditioning, respectively. The crickets were subjected to 8 pairing trials for appetitive conditioning and 12 pairing trials for aversive conditioning. The ITI was 5 min.

### Pattern preference test

The procedure for the visual pattern preference test was described previously [14]. All groups of animals were subjected to preference tests before and after conditioning. Two white-center patterns and one black-center pattern were presented on a grey sliding wall at the end of the test chamber, and two of the three patterns could be presented at the same time. A cricket was transferred to the waiting chamber and left for 4 min. Then the cricket was allowed to enter the test chamber and the test started. Then two min later, the relative positions of the black-center and white-center patterns were changed by sliding the wall. The test lasted for 4 min. If the total visiting time was less than 10 sec, we considered that the animal was less motivated to visit patterns and the data were rejected. Groups in which a black-center or white-center pattern was used as CS exhibited no significantly different levels of conditioning effect, and thus the data from the two groups were pooled.

### Second-order conditioning procedure

For achieving second-order conditioning, groups of animals were subjected to CS1-US pairing trials and then CS2-CS1 pairing trials (see Figure 5A). Pineapple or apple odor was used as CS1 and water or sodium chloride solution was used as US in appetitive or aversive second-order conditioning, respectively. For CS2, a black-center or white-center pattern was used. At the first conditioning stage, pineapple or apple odor was presented to the animal for 2 s and then a drop of water or sodium chloride solution was given to the mouth. At the second conditioning stage, CS1 and CS2 were presented at the same time for 5 s. In all experiments except for those for which results are shown in Additional file 3, animals were subjected to four CS1-US pairing trials and then four CS2-CS1 pairing trials for appetitive second-order conditioning and subjected to six CS1-US pairing trials and then four CS2-CS1 pairing trials for aversive second-order conditioning. In experiments to test the effects of drug injection on memory retention [see Additional file 3], animals were subjected to four CS1-US pairing trials and then six CS2-CS1 pairing trails for appetitive second-order conditioning and subjected to six CS1-US pairing trials and then eight CS2-CS1 pairing trails for aversive second-order conditioning. The ITI was 5 min. The intervals between the first and the second stages were either 30 min or 1 day, according to the experimental design. One control group was subjected to unpaired presentations of CS1 and US and then paired presentations of CS2 and CS1 (Unpaired/Paired or UP/P group) and another control group was subjected to paired presentations of CS1 and US and then unpaired presentations of CS2 and CS1 (paired/unpaired or P/UP group). Unpaired presentations of CSI and US or those of CS2 and CS1 were performed in a pseudo-random sequence with an interval of 2.5 min, with the number of presentations being the same as that in paired trials. Preferences between black-center and white-center patterns were tested before the first conditioning stage and at 30 min or 1 day after completing the second conditioning stage. Groups in which a black-center or white-center pattern was used as CS2 exhibited no significantly different level of second-order conditioning effects, and thus the data from the two groups were pooled.

### Pharmacology

Groups of animals subjected to (first-order) conditioning were each injected with 3 μl of physiological saline [26] or saline containing 1 μM epinastine, 1 μM mianserin, 500 μM fluphenazine, 500 μM chlorpromazine, or 500 μM spiperone into the head hemolymph at 30 min before the retention test. The estimated final concentrations after diffusion were 3.5 nM for epinastine and mianserin and 1.8 μM for fluphenazine, chlorpromazine, and spiperone, calculated from the injected volume and the approximate body weight of 850 mg. All drugs were purchased from Sigma (Tokyo, Japan). For the study using second-order conditioning, other groups of animals were each injected with 3 μl of saline or saline containing 1 μM epinastine or 500 μM fluphenazine at 30 min before the first conditioning stage, before the second conditioning stage or before the final test in second-order conditioning. The timing of injection and the concentrations of drugs used are based on our previous study [13-15]. All drugs were purchased and they were dissolved and injected by the procedure described previously [13,14].

### Data analysis

An odor or a pattern was considered to have been visited when the cricket probed it with its mouth or pulpi. The time spent visiting each odor or pattern was measured cumulatively. In appetitive conditioning, relative preference of each animal was determined using the preference index (PI) for rewarded odor or pattern, defined as *t*_*r*_/(*t*_*r*_+*t*_*nr*_) × 100, where *t*_*r *_was the time spent exploring the odor or pattern associated with reward and *t*_*nr *_was the time spent exploring the odor or pattern not associated with reward. In aversive conditioning, relative preference was determined using the PI for unpunished odor or pattern, defined as *t*_*np*_/(*t*_*np*_*+t*_*p*_) × 100, where *t*_*np *_was the time spent exploring the odor or pattern not associated with punishment and *t*_*p *_was the time spent exploring the odor or pattern associated with punishment. Wilcoxon's test (WCX test) was used to compare preferences before and after training, and the Mann-Whitney test (M-W test) was used to compare preferences of different groups. We found no significant differences in pre-conditioning odor or visual pattern preferences among different groups of animals (Kruskal-Wallis test, *P *> 0.5).

## Abbreviations

CS: conditioned stimulus; CR: conditioned response; DA: dopamine; ITI: inter-trial interval; M-U model: Mizunami-Unoki model; M-W test: Mann-Whitney test; OA: octopamine; PI: preference index; P/UP: paired/unpaired; S-R: stimulus-response; S-S: stimulus-stimulus; US: unconditioned stimulus; UR: unconditioned response; WCX test: Wilcoxon's test.

## Authors' contributions

MM conceived, designed and supervised the study and wrote the manuscript. MM and SU conceived the model. SU, YM, DH and AH carried out the experiments. YM supervised the experiments. All authors participated in the discussion and approved the final manuscript.

## Supplementary Material

Additional file 1 **An octopamine or dopamine receptor antagonist does not impair maintenance of appetitive or aversive 1-day olfactory memory, respectively**. Four groups of animals were subjected to two-trial appetitive or six-trial aversive olfactory conditioning **(A) **or eight-trial appetitive or twelve-trial aversive visual conditioning **(B)**. At 1 day after conditioning, they were each injected with 3 μl of saline containing 1 μM epinastine or 500 μM fluphenazine at 1 day before the final test. Preference indexes for rewarded odor (in the case of appetitive conditioning) or unpunished control odor (in the case of aversive conditioning) before (white bars) and after conditioning (black bars) are shown with means + SEM. The number of animals is shown at each data point. The results of statistical comparison before and after conditioning (Wilcoxon's test) are shown as asterisks (** *P *< 0.01). All groups exhibited significant levels of conditioning effects at 1 day after drug injection, at which time the drug had been fully metabolized, thus indicating that injection of epinastine or fluphenazine does not impair maintenance of appetitive or aversive memory, respectively. Click here for file

Additional file 2 **An octopamine or dopamine receptor antagonist impairs recall, but not maintenance, of appetitive or aversive 1-hour olfactory memory, respectively**. **(A) **Six groups of animals were each subjected to two-trial appetitive or six-trial aversive olfactory conditioning. At 30 min after conditioning, they were each injected with 3 μl of saline or saline containing 1 μM epinastine or 500 μM fluphenazine. Their odor preferences were tested at 30 min after injection. **(B) **Four groups of animals were each subjected to two-trial appetitive or six-trial aversive olfactory conditioning. At 30 min after conditioning, they were each injected with 3 μl of saline or saline containing 1 μM epinastine or 500 μM fluphenazine. Their odor preferences were tested at 1 day after injection. Preference indexes for rewarded odor (in the case of appetitive conditioning) or unpunished control odor (in the case of aversive conditioning) before (white bars) and after conditioning (black bars) are shown with means + SEM. The results of statistical comparison before and after conditioning (Wilcoxon's test) are shown as asterisks (*P < 0.05; **P < 0.01; NS *P *> 0.05). Epinastine fully impaired appetitive 1-h memory recall, but it had no effect on aversive 1-h memory recall. In contrast, fluphenazine fully impaired aversive 1-h memory recall, but it had no effect on appetitive 1-h memory recall. Injection of these drugs did not impair maintenance of memory, because no impairment of memory was observed when the final test was performed at 1 day after injection, at which time the drug had been fully metabolized. Click here for file

Additional file 3 **An octopamine or dopamine receptor antagonist does not impair maintenance of memory after second-order conditioning**. One group of animals was subjected to appetitive second-order conditioning with four first-stage trials and six second-stage trials, and 30 min later the group was injected with 3 μl of saline containing 1 μM epinastine. The final test was performed at 1 day after injection. The other group of animals was subjected to aversive second-order conditioning with six first-stage trials and eight second-stage trials, and 30 min later the group was injected with 3 μl of saline containing 500 μM fluphenazine. The final test was performed at 1 day after injection. Preference indexes for rewarded pattern (in the case of appetitive conditioning) or unpunished control pattern (in the case of aversive conditioning) before (white bars) and after conditioning (black bars) are shown with means + SEM. The results of statistical comparison before and after conditioning (Wilcoxon's test) are shown as asterisks (* *P *< 0.05; **P < 0.01). Both groups exhibited significant levels of second-order conditioning effects, indicating that injection of these drugs did not impair memory maintenance. Click here for file
