# Supplementary figures and images for: Roles of octopaminergic and dopaminergic neurons in appetitive and aversive memory recall in an insect
Source: BMC Biol. 2009 Aug 4;7:46. doi: 10.1186/1741-7007-7-46 (PMC2729297; doi:10.1186/1741-7007-7-46)

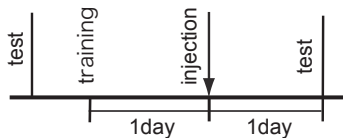

A

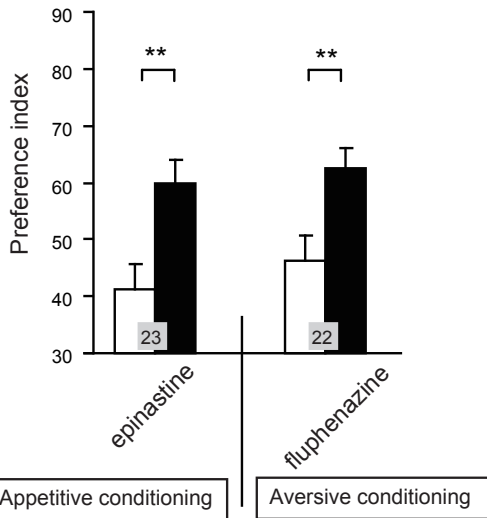

B

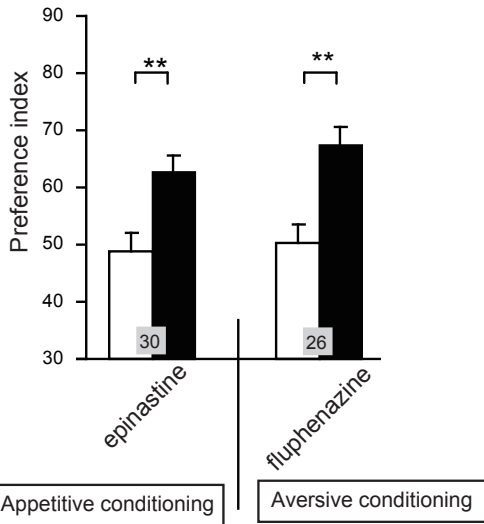

Supplement: Additional file 1 — An octopamine or dopamine receptor antagonist does not impair maintenance of appetitive or aversive 1-day olfactory memory, respectively. Four groups of animals were subjected to two-trial appetitive or six-trial aversive olfactory conditioning (A) or eight-trial appetitive or twelve-trial aversive visual conditioning (B). At 1 day after conditioning, they were each injected with 3 μl of saline containing 1 μM epinastine or 500 μM fluphenazine at 1 day before the final test. Preference indexes for rewarded odor (in the case of appetitive conditioning) or unpunished control odor (in the case of aversive conditioning) before (white bars) and after conditioning (black bars) are shown with means + SEM. The number of animals is shown at each data point. The results of statistical comparison before and after conditioning (Wilcoxon's test) are shown as asterisks (** P < 0.01). All groups exhibited significant levels of conditioning effects at 1 day after drug injection, at which time the drug had been fully metabolized, thus indicating that injection of epinastine or fluphenazine does not impair maintenance of appetitive or aversive memory, respectively. [file 1741-7007-7-46-S1.pdf]

A

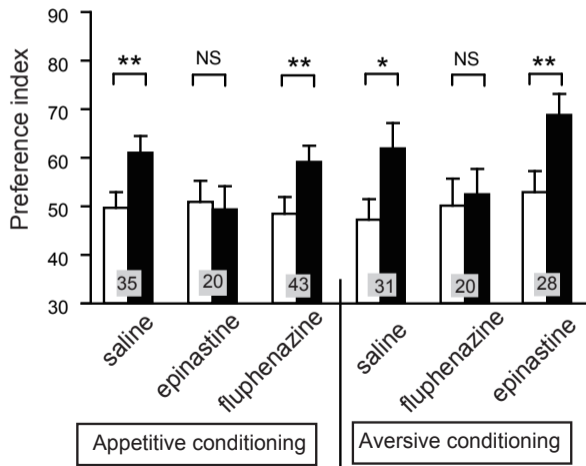

B

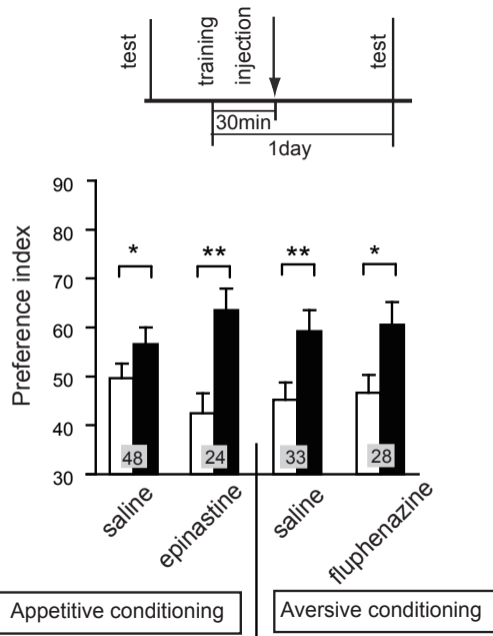

Supplement: Additional file 2 — An octopamine or dopamine receptor antagonist impairs recall, but not maintenance, of appetitive or aversive 1-hour olfactory memory, respectively. (A) Six groups of animals were each subjected to two-trial appetitive or six-trial aversive olfactory conditioning. At 30 min after conditioning, they were each injected with 3 μl of saline or saline containing 1 μM epinastine or 500 μM fluphenazine. Their odor preferences were tested at 30 min after injection. (B) Four groups of animals were each subjected to two-trial appetitive or six-trial aversive olfactory conditioning. At 30 min after conditioning, they were each injected with 3 μl of saline or saline containing 1 μM epinastine or 500 μM fluphenazine. Their odor preferences were tested at 1 day after injection. Preference indexes for rewarded odor (in the case of appetitive conditioning) or unpunished control odor (in the case of aversive conditioning) before (white bars) and after conditioning (black bars) are shown with means + SEM. The results of statistical comparison before and after conditioning (Wilcoxon's test) are shown as asterisks (*P < 0.05; **P < 0.01; NS P > 0.05). Epinastine fully impaired appetitive 1-h memory recall, but it had no effect on aversive 1-h memory recall. In contrast, fluphenazine fully impaired aversive 1-h memory recall, but it had no effect on appetitive 1-h memory recall. Injection of these drugs did not impair maintenance of memory, because no impairment of memory was observed when the final test was performed at 1 day after injection, at which time the drug had been fully metabolized. [file 1741-7007-7-46-S2.pdf]

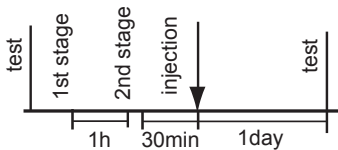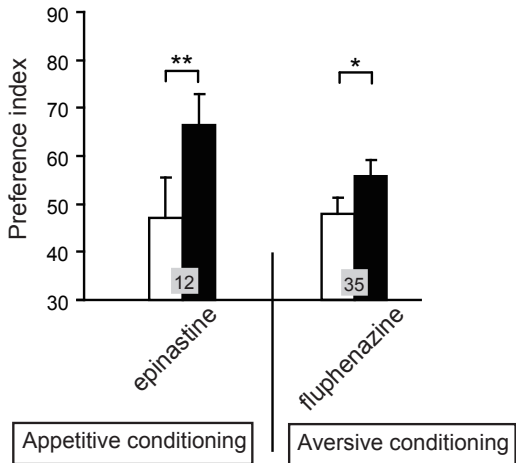

Supplement: Additional file 3 — An octopamine or dopamine receptor antagonist does not impair maintenance of memory after second-order conditioning. One group of animals was subjected to appetitive second-order conditioning with four first-stage trials and six second-stage trials, and 30 min later the group was injected with 3 μl of saline containing 1 μM epinastine. The final test was performed at 1 day after injection. The other group of animals was subjected to aversive second-order conditioning with six first-stage trials and eight second-stage trials, and 30 min later the group was injected with 3 μl of saline containing 500 μM fluphenazine. The final test was performed at 1 day after injection. Preference indexes for rewarded pattern (in the case of appetitive conditioning) or unpunished control pattern (in the case of aversive conditioning) before (white bars) and after conditioning (black bars) are shown with means + SEM. The results of statistical comparison before and after conditioning (Wilcoxon's test) are shown as asterisks (* P < 0.05; **P < 0.01). Both groups exhibited significant levels of second-order conditioning effects, indicating that injection of these drugs did not impair memory maintenance. [file 1741-7007-7-46-S3.pdf]
